# A scheme for 3-dimensional morphological reconstruction and force inference in the early *C. elegans* embryo

**DOI:** 10.1371/journal.pone.0199151

**Published:** 2018-07-10

**Authors:** Muzhi Xu, Yicong Wu, Hari Shroff, Min Wu, Madhav Mani

**Affiliations:** 1 Engineering Sciences and Applied Mathematics, Northwestern University, Evanston, Illinois 60208, United States of America; 2 Section on High Resolution Optical Imaging, NIBIB, NIH, Bethesda, Maryland 20892, United States of America; 3 Mathematical Sciences, Worcester Polytechnic Institute, Worcester, Massachusetts 01609, United States of America; 4 Molecular Biosciences, Northwestern University, Evanston, Illinois 60208, United States of America; University of California Santa Barbara, UNITED STATES

## Abstract

In this study, we present a scheme for the reconstruction of cellular morphology and the inference of mechanical forces in the early *C. elegans* embryo. We have developed and bench-marked a morphological reconstruction scheme that transforms flourescence-based *in vivo* images of membranes into a point cloud of smoothed surface patches, which facilitates an accurate estimation of membrane curvatures and the angles between membranes. Assuming an isotropic and homogeneous distribution of tensions along individual membranes, we infer a pattern of forces that are 7% deviated from force balance at edges, and 10% deviated from the Young-Laplace relation across membranes. We demonstrate the stability of our inference scheme via a sensitivity analysis, and the reproducibility of our image-analysis and force inference pipelines.

## Introduction

The emergence of morphology during organismal development, morphogenesis, consists of an interplay between biochemical signaling and mechanical forces. In particular, mechanical forces are a consequence of the states of cells in the embryo and determine the geometry, future positions and contact-map of cells [1, 2]. Furthermore, the prevalence of mechanotransduction in biological system [3, 4] suggests that the map between cell-fate and mechanical state can be bidirectional. Despite our acquisition of an ever growing list of participating molecular factors, the collective nature of morphogenetic processes precludes a straightforward genotype-to-phenotype map. Understanding details of this map will help advance our understanding of morphogenesis.

Live fluorescence-based imaging facilitates pursuit of a phenomenological approach to morphogenesis by giving us the ability to quantify the geometry of cellular shapes and flows, and the dynamics of the cytoskeleton. However, given the complexity of a cell’s material, likely time-dependent, properties, it is challenging to infer mechanical stresses from observed patterns of deformations and flows. In short, the tools necessary to accurately and robustly measure the forces that determine cellular geometries and drive cellular flows in embryos are in their infancy. Recently, a set of image-analysis based indirect force inference schemes have begun to produce maps of forces in quasi two-dimensional epithelial tissues [5–11]. Force inference schemes are based on the assumptions of force balance and are constructed from the geometry and flow of the tissue alone. The assumption of a non-dissipative force balance is justified in settings where the timescales associated with cellular motion are large compared to the relaxation timescales observed following laser ablation events. This juxtaposes approaches that account for velocity data [8, 9, 12], which necessarily require further material assumptions. Pursuing such an approach, the inferred cell-cell contact forces have been shown to correlate well with the average line density of molecular motor distributions [10, 13] in 2D epithelia. It is worth noting that while the emergence of FRET-based force reporters is exciting, connecting molecular-scale loads to the macroscopic forces that drive morphogenetic movements is a challenge [14].

Recent advances in the live-imaging of the *C. elegans* embryo gives unprecedented access to the 3D geometry and dynamics [15–18] of the small number of cells as they make important decisions in the life of the worm [19]. In this study, we present novel schemes for the reconstruction of cellular morphology and the inference of forces in the early *C. elegans* embryo. In particular, we present details of 1) an image analysis protocol that produces high-resolution reconstruction of membrane and junction geometry, and 2) a scheme that gives access to the relative membrane tensions and cellular pressures in the *C. elegans* embryo. The enhanced accuracy of our morphological reconstruction is essential for inference of forces. Assuming an isotropic and homogeneous distribution of tensions along a membrane, we infer a pattern of forces that are 7% away from force balance at junctions, and 10% away from the Young-Laplace relation at membranes. We present a sensitivity analysis that demonstrates the stability of our scheme. Lastly, we confirm that the reproducibility in the image-analysis pipeline is on the order of 5%. The quantitative assessment of the methodology presented in this study suggests improvements that we comment upon in our discussions section, and will guide future projects.

## Methods

### Force balance relations

We assume that the mechanical state of cells in the early worm embryo is dominated by intracellular pressures and intercellular membrane tensions. For each cell, indexed *c*, we define a pressure *P*_*c*_. For each membrane, indexed *m*, we define a tension *T*_*m*_. From a single image we infer the unknown parameters $\left(P_{1},\ldots ,P_{n_{c}},T_{1},\ldots ,T_{n_{m}}\right)$ where *n*_*c*_ is the number of cells and *n*_*m*_ is the number of membranes. Importantly, we ignore dissipative forces associated with the dynamics of the embryo underpinning our neglect of velocity data.

**Fig 1:**
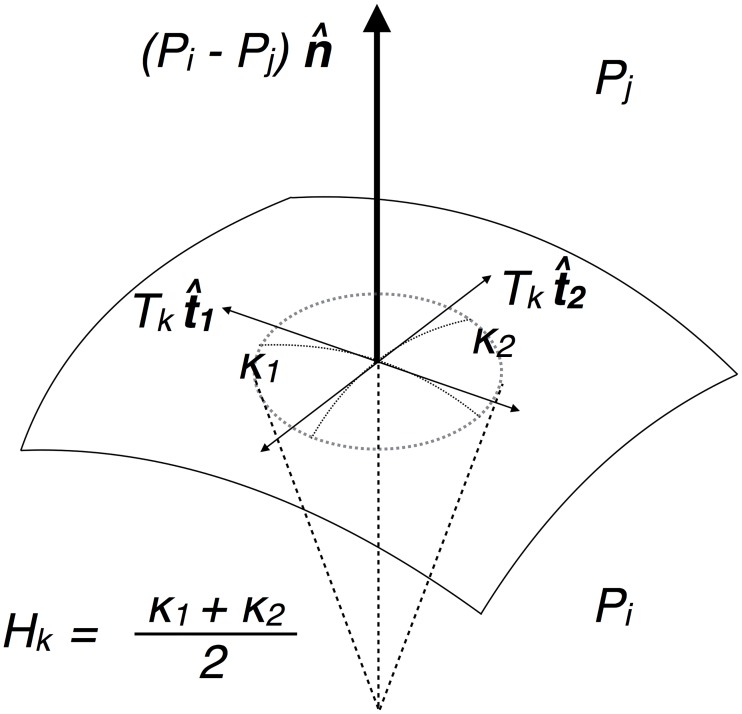
Schematic for the Young-Laplace relation on a membrane. The schematic depicts a small membrane patch, where the normal pressure force on the membrane is balanced locally by the surface tension. Assuming isotropic tensions, the Young-Laplace relation, *P*_*i*_ − *P*_*j*_ = 2*H*_*k*_*T*_*k*_, is characterized by the mean curvature $H_{k}=\frac{\kappa_{1}+\kappa_{2}}{2}$—the two principle curvatures are $\kappa_{1,2}=-n\cdot \frac{d{\hat{t}}_{1,2}}{ds}$, where **n** is the normal.

**The Young-Laplace relation**: We employ the Young-Laplace relation on each membrane that relates the jump in pressure across a membrane to the product of its mean curvature and tension. The use of the Young-Laplace relation rests on the assumption that the membrane is fluid-like. We neglect inhomogeneities and anisotropies in tensions along a membrane, which is tantamount to assuming that the variation of tension along a membrane is negligible compared to its average. The following relation

$$\begin{array}{c} P_{i}-P_{j}=2\cdot H_{k}\cdot T_{k} \end{array}$$ (1)

holds for each membrane face *k* (with adjacent cells *i* and *j*) where *P*_*i*_ and *P*_*j*_ are the pressures of cells *i* and *j*, respectively. *H*_*k*_ and *T*_*k*_ correspond to the mean curvature and tension of face *k*, respectively (See Fig 1). We have *n*_*m*_ Young-Laplace relations, where the mean curvature *H*_*k*_ is obtained by taking the average of the mean curvatures along the membrane. More details are given in the *Image Analysis Protocol* section.

**Fig 2:**
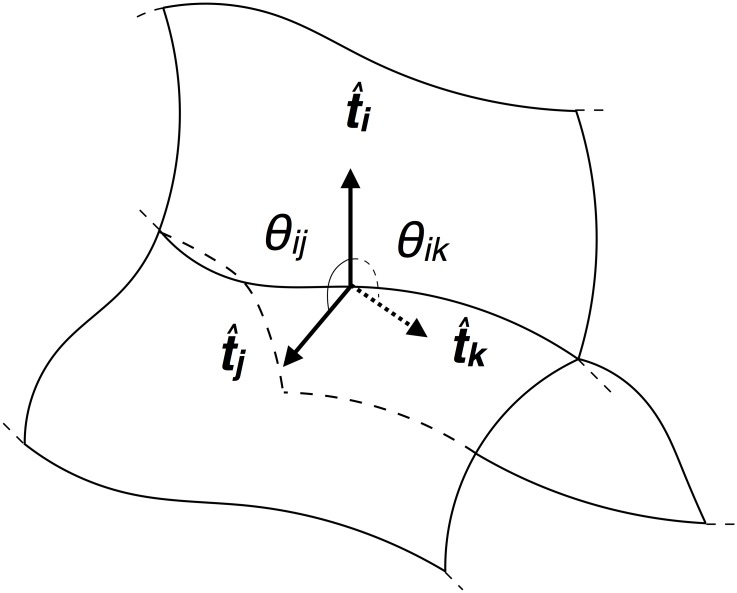
Schematic for tension balance at a junction. The three membrane faces, illustrated as curved planes, intersect at a junction. The isotropic surface tensions act perpendicular to the junction. The dihedral angles of intersection between the faces prescribe the relation in Eqs (2) and (3).

**Junctional force balance**: Curvilinear junctions are formed by the intersection of three membranes. At every point along these curvilinear junctions we assume that the tension from the three intersecting membranes should balance each other (See Fig 2). Mathematically, the tensions *T*_*i*_, *T*_*j*_ and *T*_*k*_ are related by $T_{i}{\hat{t}}_{i}+T_{j}{\hat{t}}_{j}+T_{k}{\hat{t}}_{k}=\vec{0}$ where ${\hat{t}}_{i}$, ${\hat{t}}_{j}$, and ${\hat{t}}_{k}$ are the unit vectors normal to the junction, and tangent to the membrane surfaces *i*, *j*, and *k* respectively. The vector equation can be rewritten as

$$\begin{array}{cc} T_{i}+T_{j}\cos\theta_{ij}+T_{k}\cos\theta_{ik} & =0 \end{array}$$ (2)

$$\begin{array}{cc} T_{j}\sin\theta_{ij}-T_{k}\sin\theta_{ik} & =0 \end{array}$$ (3)

where *θ*_*ij*_ (*θ*_*ik*_) is the dihedral angle between ${\hat{t}}_{i}$ and ${\hat{t}}_{j}$ (${\hat{t}}_{i}$ and ${\hat{t}}_{k}$), respectively. In principle, this relation holds at every point along the junction as the local tensions and angles vary. In this work, we define a single value for tension on each membrane, giving us a single equation for each junction with constant *T*_*i*_, *T*_*j*_ and *T*_*k*_ − *θ*_*ij*_ and *θ*_*ik*_ are taken as the averages along a junction.

**Solving the system of equations**: We define the vector $x={\left(P_{0},P_{1},\ldots ,P_{n_{c}},T_{1},\ldots ,T_{n_{m}}\right)}^{T}$ where *P*_0_ is the constant pressure exterior to the embryo, $P_{1},\ldots P_{n_{c}}$ are the cellular pressures and $T_{1},\ldots ,T_{n_{m}}$ are the membrane tensions. We can solve the linear system

$$\begin{array}{c} Mx=b. \end{array}$$

for the pressures and tensions, where the size of **M** is (*n*_*m*_ + 2 × *n*_*j*_ + 2) × (*n*_*m*_ + *n*_*c*_ + 1). The first *n*_*m*_ rows of **M** correspond to the force balance relations along the membrane (Eq (1)), and the next 2 × *n*_*j*_ rows correspond to the force balance relations along the junctions (Eqs (2) and (3)). The two additional rows house two equations that 1) fix the exterior pressure, *P*_0_ = *P*_*b*_, and 2) set the scale of tensions. We scale the average tension to be $1-\Sigma_{i=1}^{n_{m}}T_{i}=n_{m}$. Finally, we have **b** = (0, …, 0, *P_b_*, *n_m_*)*^T^*. Notice that the relation between the tension and pressure from the Laplace’s Equation depends on the length scale of the image since they hold different units. In practice, we can set a tension scale and length scale, which effectively determines the pressure scale. Based on the inequality *n*_*m*_ + *n*_*c*_ + 1 ≤ *n*_*m*_ + 2 × *n*_*j*_ + 2 (can be proved by induction from a single cell, where 1 + 1 + 1 ≤ 1 + 2 × 0 + 2 holds), the system is overdetermined and we can only solve **x** in the sense of minimization of the error ‖**Mx** − **b**‖^2^. This is equivalent to $x=\tilde{M}b$ where $\tilde{M}={\left(M^{T}M\right)}^{-1}M^{T}$ is the pseudoinverse of **M**. When **M** is full-column-rank, which is always the case in our practice, the corresponding pseudoinverse problem has a unique solution. When **M** is full-column-rank, which is always the case in our practice, the corresponding pseudoinverse problem has a unique solution.

### Imaging

Nematode strain BV24 ([ltIs44 [pie-1p-mCherry::PH(PLC1delta1) + unc-119(+)]; zuIs178 [(his-72 1kb::HIS-72::GFP); unc-119(+)] V]) was used for membrane imaging. ltIS44 is an integrated transgene which expresses membrane-localized mCherry; zuIS178 is an integrated transgene that expresses histone-GFP fusion, which was not imaged in this study. Worms were raised under standard conditions at 20°C on NGM media seeded with E. coli OP50. The embryo was transferred to rectangular coverslips (VWR, 48393-241) and then placed into an imaging chamber (Applied Scientific Instrumentation, I-3078-2450) as previously described. The embryo was imaged with dual-view inverted selective-plane illumination microscopy (diSPIM)2, although only one view was selected for analysis. The illumination wavelength was 561 nm (Crystalaser, CL-561-050), mCherry fluorescence was collected via the 0.8 NA detection objective (Nikon 40×, 3.5 mm working distance, water immersion lens) transmitted through dichroic mirrors (Chroma, ZT405/488/561rpc), filtered through a notch emission filters (Semrock, NF03-561E-25) to reject the 561-nm pump light, and imaged with 200-mm tube lenses (Applied Scientific Instrumentation, C60-TUBE_B) onto scientific-grade, complementary, metal-oxide semiconductor (sCMOS) cameras (PCO, Edge). The resulting image pixel size was 6.5 *μ*m/40 = 162.5 nm. We recorded 80 planes per volume for the embryo, 5 ms per plane, spacing planes every 0.5 *μ*m. Volumes were recorded at a temporal resolution of 1 min from 4-cell stage until hatching (i.e., 13 hours post fertilization).

### Morphological reconstruction of the embryo

The coefficient matrix **M** holds the averaged mean curvatures *H*_*k*_ of the k-th membrane and the averaged dihedral angle *θ*’s between intersecting membranes. Before we compute the averaged curvatures and angles, we need to reconstruct these parameters locally, at each point along the membrane and junction. These geometric quantities, especially the curvatures *κ*_*m*_, involve the second-order derivatives of position vectors along the membrane surfaces that are sensitive to noise, which can be introduced at every stage leading up to an image.

**Fig 3:**
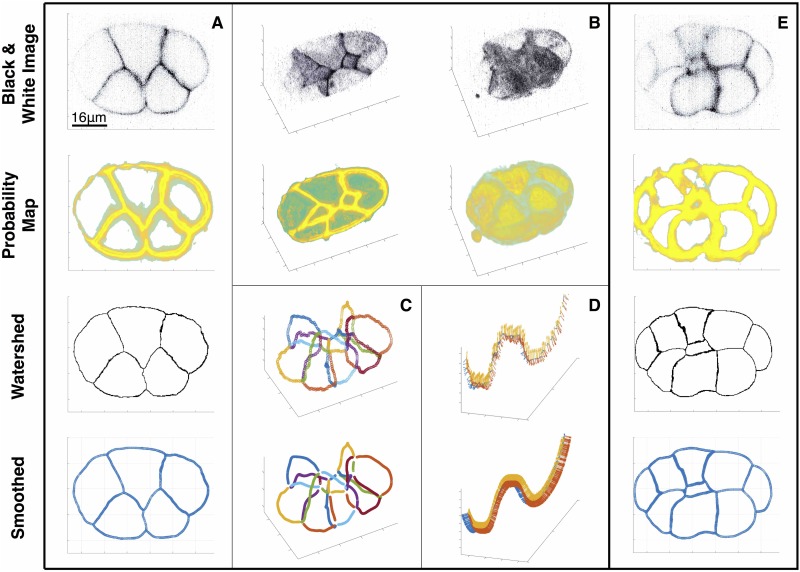
Workflow of embryo reconstruction. (**A**) Shows a cross-sectional grayscale image of a 7-cell embryo, the ilastik outputted probability map, and a smoothed point cloud representation. (**B**) 3D visualization of the binarized image and probability map, with a cross section (left) and the whole embryo (right). (**C**) Plot of all the junctions in an embryo. The effect of smoothing can be seen clearly here. (**D**) Depiction of a junction, along with the tangent vectors of the adjacent membranes. The sparser watershed-based junction is replaced by a denser and more robust point cloud representation. (**E**) shows the cross-sectional grayscale image of a 12-cell embryo, the probability map, and a smoothed point cloud representation.

Instead of working with the gray-scale image itself, we work with the probability map generated by the pixel classification scheme of the machine learning image analysis software (*Ilastik*)—a nonlinear transform of the underlying data based on supervised training. See S1 Text for more details. We show two samples of the grayscale image and the probability map in Fig 3(A), 3(B) and 3(E), where the first two rows in panel A, E and B show the the cross-sectional image of the 7-cell-embryo and 12-cell-embryo, together with 3D image of the 7-cell-embryo, respectively. The third rows in panel A and E show a cross section of the segmented one-voxel-thick membrane structure. The reconstructed membranes are artificially distorted or flattened due to their restriction on the voxel mesh. The distortion can result in an unreliable estimate of the normal vectors and in an inaccurate computation of the curvatures along the membrane’s surface. Moreover, the junctions reconstructed by taking the intersection of three membrane surfaces are also distorted and can disrupt the computation of the tangent vectors of the junctions and the angles between intersecting membranes. See the first row of panel D in Fig 3 of the disorganized tangent vectors.

To deal with this artifact, we smooth the membrane surfaces based on a principal component analysis (PCA) of the membrane points obtained by the watershed transformation. The smoothed membrane surfaces are reconstructed on a three-dimensional point cloud, a collection of mesh-free points. The outcome of this protocol can be seen in the fourth row of panel A and the second row of panel D from Fig 3, compared with their unsmoothed counterparts above. The details of this method are discussed in the section, *Point cloud normal estimates and smoothing*. Based on the smoothed membranes and junctions, we compute the curvatures and the angles between intersecting membranes, detailed in *The curvatures of the membrane surfaces* and *The angles between membranes along the junctions*, respectively.

**Point cloud normal estimates and smoothing**: The surface normal vectors are required to compute the curvatures and the angles between the intersecting membrane surfaces. We estimate the surface normal [20, 21], which smooths the surface locally and estimates the normal simultaneously. Given that the surface is sufficiently smooth, the surface normal at a point **p** can be obtained by finding the unit vector ‖***η***‖ = 1, which minimizes $\sum_{k=1}^{K}{\left(\left(x_{k}-p\right)\cdot \eta \right)}^{2}$, where **x**_*k*_’s are the *K*-nearest neighboring points of **p** (see *K*_*S*_ in the S2 Table). This approach is equivalent to the normal estimate method from principal component analysis (PCA). In order to smooth the surface, a shift *t* is introduced [20, 21] along the unknown normal direction ***η***, and the constrained least square problem can be reformulated to: Find *t* and ***η*** that minimize

$$\begin{array}{c} \min_{\eta ,t}\sum_{k=1}^{K}{\left(\left(x_{k}-\left(p+t\eta \right)\right)\cdot \eta \right)}^{2}\;\text{where}\;\|\eta \|=1. \end{array}$$

at the shifted position $\tilde{p}=p+t\eta$. Notice the computation of ***η*** at each point only needs information from the *K*-nearest neighboring points, instead of all membrane points. After computing the surface normal vectors at every point along the surface, there is no guarantee that their orientation will be consistent. To address this we propagate the consistent direction of the normal vectors along the Euclidean minimum spanning tree that connects the points [20–22].

**Estimating membrane curvatures**: We calculate the mean curvature *κ*_*m*_(**p**) at each membrane point **p** = (*x*_*p*_, *y*_*p*_, *z*_*p*_) by first parametrizing the membrane patch including the K-nearest neighboring points **x***_k_* = (*x*_*k*_, *y*_*k*_, *z*_*k*_)’s based on the normal estimate ***η*** and smoothing from the last section. At each point, we define a local *u* − *v* − *z* cartesian coordinate system where *u* and *v* span the tangent plane and *z* is along the normal direction ***η***. Then we parametrize the surface patch by **r**(*u*, *v*) = *u***t**_1_ + *v***t**_2_ + *z*(*u*, *v*)***η***, where **t**_*i*_’s are the unit tangent vectors. *z*(*u*, *v*) can be approximated by fitting the the local surface by a second order Taylor expansion about **p**, minimizing

$$\begin{array}{c} \min_{a,b,c,d,e}\;\sum_{k=1}^{K_{S}}{\left[\;-{\tilde{z}}_{k}+a{\tilde{x}}_{k}+b{\tilde{y}}_{k}+\frac{c}{2}{\tilde{x}}_{k}^{2}+d{\tilde{x}}_{k}{\tilde{y}}_{k}+\frac{e}{2}{\tilde{y}}_{k}^{2}\;\right]}^{2}. \end{array}$$ (4)

where ${\tilde{z}}_{k}$’s are the projections of (**x**_*k*_ − **x**_*p*_)’s along ***η***, and ${\tilde{x}}_{k}$’s and ${\tilde{y}}_{k}$’s are their projections in the tangent plane. Then the curvatures can be computed from the shape operator *W*_*p*_, which is a 2 × 2 matrix (also called Weingarten matrix) where the components can be calculated by differentiating **r**(*u*, *v*) up to the second order. See more details in S1 Text. The local mean curvature *κ*_*m*_ is calculated by taking the average of the two eigenvalues of the matrix (principal curvatures). Notice the computation of *κ*_*m*_(**p**) at each point only needs information from the *K*-nearest neighboring points, instead of all membrane points. We also further update the normal $\eta \to \eta -\frac{a}{\sqrt{a^{2}+b^{2}+1}}t_{1}-\frac{b}{\sqrt{a^{2}+b^{2}+1}}t_{2}$ accordingly.

**Fig 4:**
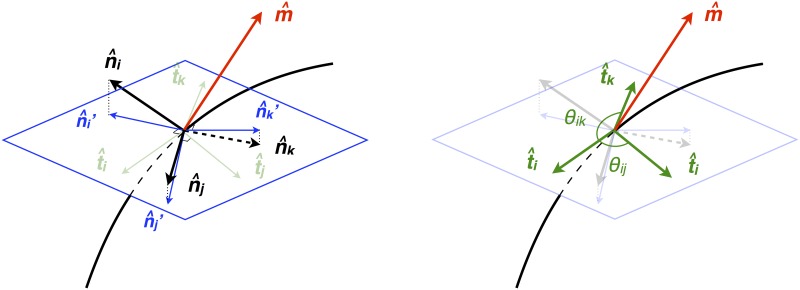
Schematic for tangent and angle computation. The black curved line represents the junction and the black arrows tagged by ${\hat{n}}_{i}$, ${\hat{n}}_{j}$, and ${\hat{n}}_{k}$ represent the normal vector of each membrane face. The red vector $\hat{m}$ is an approximation to the junction’s tangent vector. The blue plane represents the perpendicular plane to the red vector $\hat{m}$. The blue vectors are the projected normals, which lie in the plane normal to $\hat{m}$, and thus are coplanar. On the right, we demonstrate that the angle of intersections can be calculated from the tangent vectors ${\hat{t}}_{i}$, ${\hat{t}}_{j}$, and ${\hat{t}}_{k}$ from each membrane normal to the junction.

**Estimating the angles between membranes:** On points along a junction we define the local angles between the three intersecting membranes in the plane normal to the tangent direction (normal plane, see the blue plane in Fig 4). We need to 1) reconstruct the junction, 2) estimate the normal plane and 3) compute the angles.

To reconstruct the junction we sample points from each of the three intersecting membranes into a point cloud. For each of the three membranes, a membrane point is included in the junction if it is within a distance threshold *d*_*T*_ (see S2 Table) from the two adjacent membranes. Within the point cloud, we update the position of each point by the average of the position vectors of its K-nearest neighboring points (see *K*_*J*_ S2 Table). This update turns the point cloud into a thinned curve representing the junction.

To estimate the normal plane along the thinned junction, we define the tangent vector on each point along the junction curve, approximated by

$$\begin{array}{c} \hat{m}=\frac{{\hat{n}}_{1}\times {\hat{n}}_{2}+{\hat{n}}_{2}\times {\hat{n}}_{3}+{\hat{n}}_{3}\times {\hat{n}}_{1}}{\|{\hat{n}}_{1}\times {\hat{n}}_{2}+{\hat{n}}_{2}\times {\hat{n}}_{3}+{\hat{n}}_{3}\times {\hat{n}}_{1}\|}. \end{array}$$

${\hat{n}}_{1}$, ${\hat{n}}_{2}$, and ${\hat{n}}_{3}$ are the surface normals evaluated on the three closest points from the three membranes. We then define the adjusted normals ${\hat{n}}_{1}^{\prime }$, ${\hat{n}}_{2}^{\prime }$, and ${\hat{n}}_{3}^{\prime }$ by

$$\begin{array}{c} {\hat{n}}_{i}^{\prime }={\hat{n}}_{i}-\hat{m}\left(\hat{m}\cdot {\hat{n}}_{i}\right) \end{array}$$

The adjusted normals are in the normal plane, as shown in Fig 4. We can now compute surface tangents ${\hat{t}}_{i}$ orthogonal to the junction by

$$\begin{array}{c} {\hat{t}}_{i}=\hat{m}\times {\hat{n}}_{i}^{\prime } \end{array}$$

for membrane face *i*. The angles *θ*_*ij*_ and *θ*_*ik*_ from Eqs (2) and (3) are finally computed by taking the differences between ${\hat{t}}_{i}$’s.

## Results

### *In-silico* validation of the scheme

**Fig 5:**
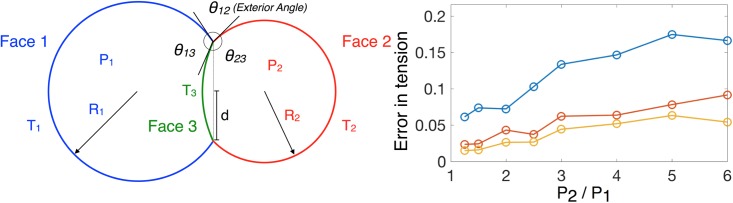
Schematics of a two-cell system and *in-silico* assesment of force inferences. On the left, the system depicts the cross-sectional slice of two cells (with pressures *P*_1_ and *P*_2_), with constant mean curvatures, *H*_1_ = 1/*R*_1_ and *H*_2_ = 1/*R*_2_, on the major membrane faces 1 and 2 (with tensions *T*_1_ and *T*_2_) separated by the interfacial membrane face 3 (with tension *T*_3_) with constant mean curvature *H*_3_. Note, all three membranes are patches of spherical membranes as they have constant mean curvatures. *d* denotes the radius of the circular junction between the 3 membrane faces. *θ*’s correspond to the angles between the 3 membranes along the circular junction. On the right, we measure the total error between the true tension and the inferred tension: $\Sigma_{i=1}^{3}\left|T_{i}-T_{i,true}\right|$.

Our model assumes that the mechanical state of the early worm embryo is dominated by intracellular pressures and intercellular membrane tensions, which are isotropic and uniform on each membrane face. We test are method against an *in-silico* two-cell systems where the above assumptions are fully satisfied. In detail, the configuration of a two-cell system can be fully determined by the tensions along the three membrane faces and pressures within the two cells. We generate a family of synthetic membrane images where the radii of the two cells (*R*_1_ = 5 × *L* and *R*_2_ = 4 × *L*) and the radius of the closed circular junction (*d* = 3 × *L*) are fixed (*L* is the length scale). See the left panel in Fig 5 for the schematics. By varying the ratio of pressures (*P*_2_/*P*_1_) and the ratio of tensions accordingly, we can maintain the radii of the two cells while changing the mean curvature of the interfacial membrane (*H*_3_). For this family of configurations with different *P*_2_/*P*_1_’s, we generate 3D images with probability maps of membrane faces with different spatial resolutions. We choose *L* = 10, 20, 40 to effectively change the resolutions. *L* = 10 corresponds to approximately 45 voxel numbers across the cell diameter while *L* = 40 corresponds to approximately 180 voxel numbers across the cell diameter. Based on the probability maps, we extract the curvatures of the three membranes 1/*R*_1_, 1/*R*_2_ and *H*_3_ and the angles *θ*_12_, *θ*_13_ and *θ*_23_ between the three membranes along the circular junction. We report that the reconstruction of the curvatures of the two major membranes 1/*R*_1_ and 1/*R*_2_ are reliable regardless of the different resolutions used (see errors between the reconstructed and true curvature in S1A Fig), while the curvature reconstruction of the interfacial membrane *H*_3_ is improved upon increase in the resolution (see S1B Fig). Notice as *P*_2_/*P*_1_ tends to 1, the curvature of the interfacial membrane *H*_3_ approaches to 0, driving an increase in relative errors as *P*_2_/*P*_1_ decreases in Fig 5(B). The reconstruction of angles *θ*_12_, *θ*_13_ and *θ*_23_ is also improved upon an increase in resolution (see S1C Fig for the total error $\frac{1}{2\pi }\Sigma \left|\theta -\theta_{true}\right|$). With respect to our force inference scheme, we show that the total relative error ($\Sigma_{i=1}^{3}\left|T_{i}-T_{i,true}\right|$) between the inferred tensions and the true tensions is below 0.2 in all cases, and decreases when the resolution increases (See the right panel in Fig 5(B)). The relative residuals of the Young-Laplace and the force balance relations (normalized as described in the results *Quantitative assessment of errors*) are below 0.02 and 2 × 10^−5^, respectively (See S1E and S1F Fig).

### Morphological reconstruction of worm embryos

**Fig 6:**
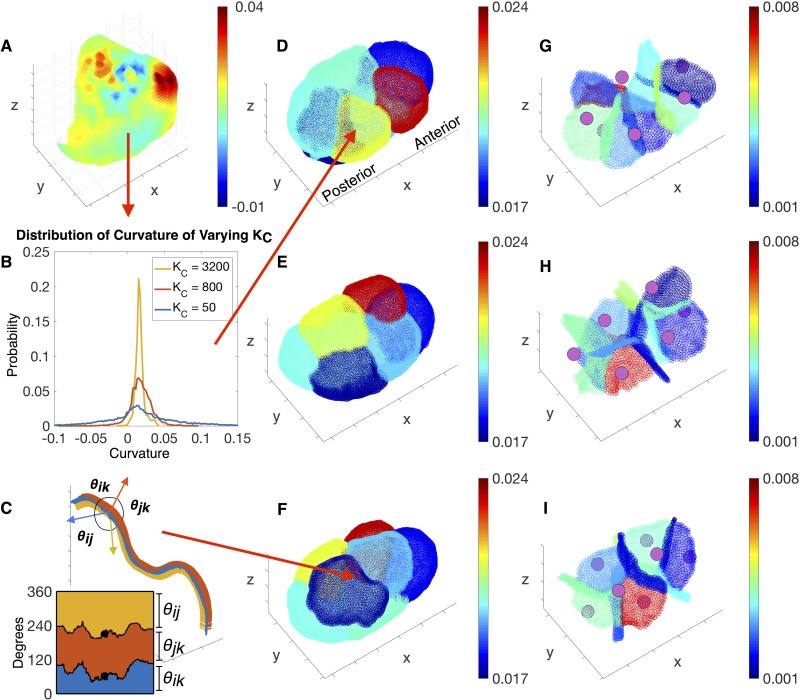
Reconstruction of curvatures and angles. (D-F) Shows the average mean curvatures *H*_*k*_’s on the exterior membranes, and (G-I) shows the average mean curvatures on the interior membranes by rotating the anterior-posterior axis by different angles. See S1 and S2 Movies for the average mean curvatures for both the 7-cell-embryo and 12-cell-embryo. The average mean curvature, *H*_*k*_, on each membrane’s is calculated by taking the average of mean curvatures *κ*_*m*_(**p**) over all points on the membrane. A shows an example of the mean curvature distributed on a single membrane face, and B shows the distribution of how mean curvatures vary when using a different number of nearest neighboring points, *K*_*C*_. The overall averaged mean curvature is not sensitive to the choice of *K*_*C*_. Henceforth, we use *K*_*C*_ = 50 (also see S2 Table) to calculate the average mean curvatures *H*_*k*_’s on each membrane. We calculate the three average angles *θ*’s along each junctions by taking the average of three angles over all points on the same junction. C shows an example of the angle variations along one junction.

See Fig 6 for the reconstructed mean curvatures *H*_*k*_’s of the membrane faces and averaged dihedral angles along the curvilinear junctions *θ*’s using the workflow *Morphological reconstruction of the embryo*. We obtain the average mean curvature *H*_*k*_ on each membrane face *k* by taking the average of local mean curvatures, *κ*_*m*_, along each membrane. See Fig 6(A) for the heat map of *κ*_*m*_ on an exterior membrane. Since the local mean curvature at a point **p** is obtained by fitting the neighboring membrane points by a paraboloid (see Eq 4 in *The curvatures of the membrane surfaces*), the result vary with *K*_*C*_, the number of nearest neighboring points used. However, the averaged mean curvature *H*_*k*_ is insensitive to the choice of *K*_*C*_. See Fig 6(B) for the distributions of mean curvatures *κ*_*m*_ using *K*_*C*_ = 50, 800 and 3200. Accordingly, we fix *K*_*C*_ = 50 to calculate the *κ*_*m*_’s and the associated *H*_*k*_’s. See Fig 6(D)–6(F) and 6(G)–6(I) for the averaged mean curvature *H*_*k*_’s on the exterior membranes and interior membranes in different views. See S1 and S2 Movies for more details of *H*_*k*_’s in both the 7-cell-embryo and 12-cell-embryo. Notice that the curvature we obtain is in units of voxel sizes, and later we directly use the non-dimensional curvature to infer relative pressures. Similarly, we calculate the averaged dihedral angles between membranes along the curvilinear junction from the local dihedral angle computations following *The angles between membranes along the curvilinear junctions*. See Fig 6(C) for the angle variations along one junction intersected between two exterior membranes and one interior membrane. With respect to our proposed force inference scheme we obtain all the parameters needed in the coefficient matrix **M** to infer forces in both a 7-cell-embryo and 12-cell-embryo.

### Force inference on the embryo

**Fig 7:**
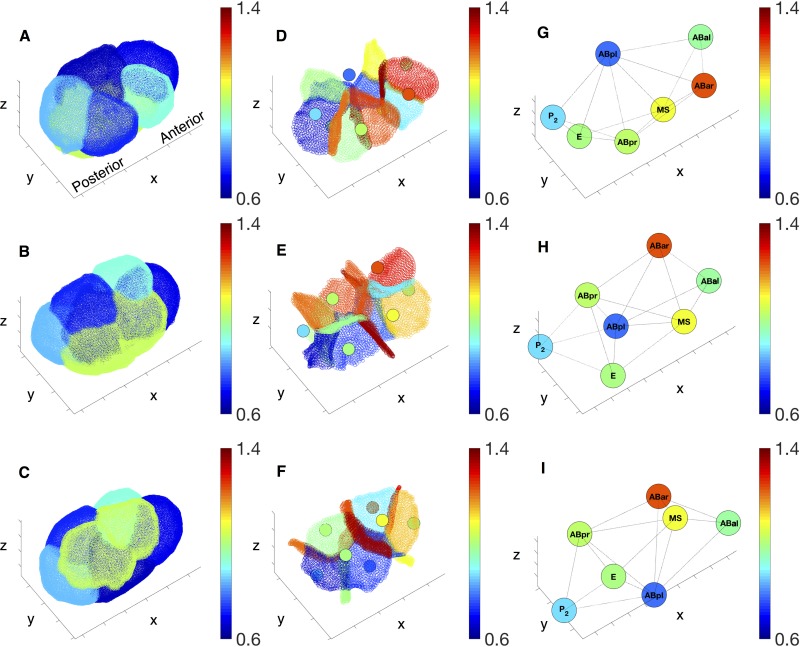
Inferred forces. Inferred tensions and pressures at the 7-cell stage. (A-C) Relative tensions on the outer membranes. (D-F) Relative tensions on the inner membrane and the relative pressures in cells (represented as colored circular region in the middle of each cell). (G-I) Pressures in the cells. In these images, all pressure values are rescaled to match the same scale as the relative tension. The three rows depict the embryo from different viewpoints.

We infer membrane tensions cellular pressures according to the model in *Force balance relations*, shown in Fig 7 from three different views. The pressures are shown in the second column (panel D-F) together with the tension map of the inner membranes, as well as in the third column (panel G-I) where each cell is labeled by its name. The ABar cell on the anterior of the embryo sustains the highest level of pressure, while the ABpl cell on the posterior sustains the lowest level of pressure. Interestingly, the P2 cell, which is about to divide at this stage in the movie, shows a relatively low level of pressure. A global monotonic anterior-posterior gradient of pressure can be discerned. In contrast, we cannot identify a similar anterior-posterior trend in membrane tensions. While the distribution of tensions on the inner membranes are heterogeneous, one can see that most of the inner membranes (panel D-F) present higher level of tensions than the outer membranes (panel A-C). We conjecture that this is likely a consequence of outer membranes only comprising approximately half the cytoskeleton activity that inner membranes posses. See S3 and S4 Movies for more details of inferred forces in both 7-cell-embryo and 12-cell-embryo. Notice that our method neglects any interactions between the egg shell and external cell membranes [23]. Furthermore, all the above trends need to be confirmed across biological replicates, and confronted to myosin density data.

**Fig 8:**
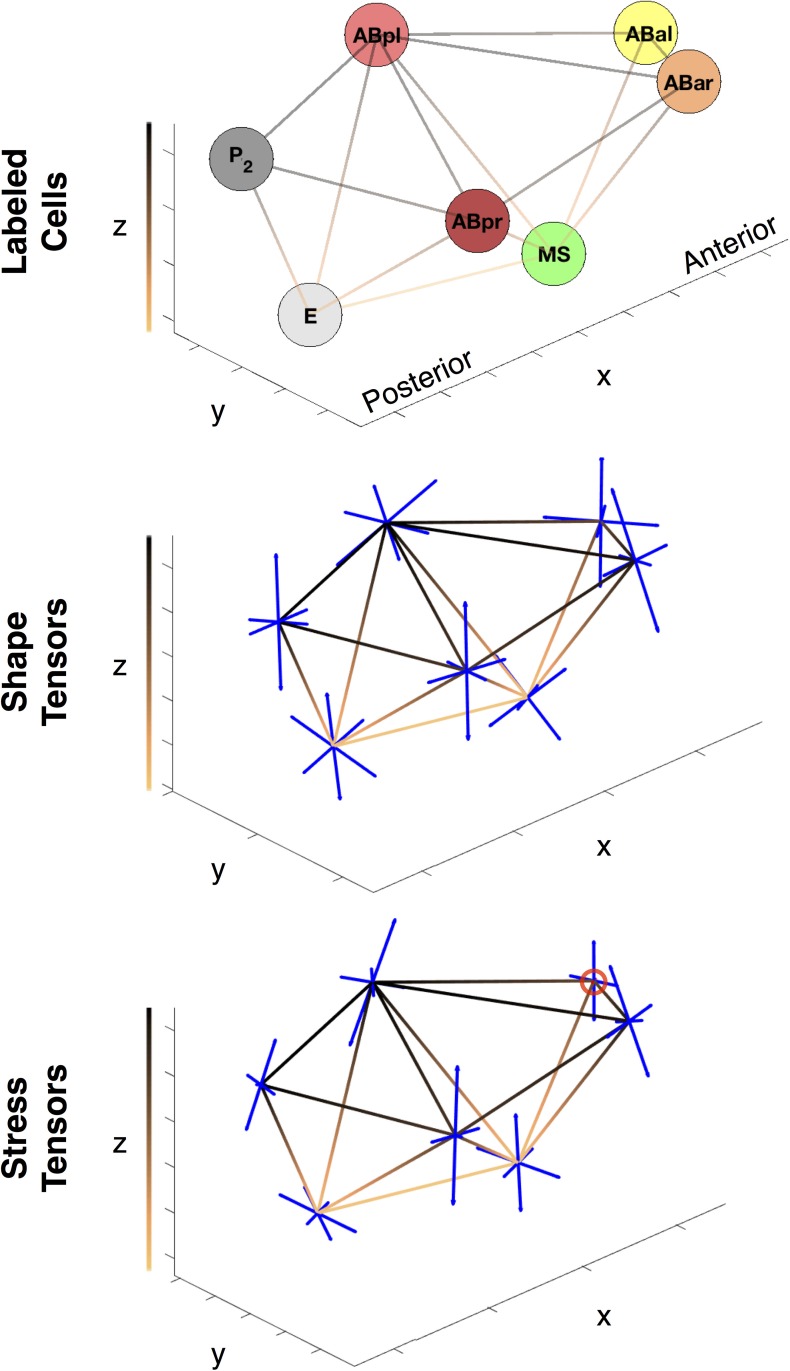
Depiction of the average mechanical state and cell-shape tensors. Plot of the shape and stress tensors at the 7-cell stage. The copper gradient lines represent cell connectivity, while the colors ranging from black to copper corresponds the depth along the z-axis. The tensors (all of which are symmetric) are represented by their 3 orthogonal eigenvectors plotted as blue line segments. The length of the segments correspond to the magnitude of the eigenvalue. Compressive forces in the stress tensors are plotted as red lines, and are circled in red for clarity.

Based on the inferred tension and pressure, we can further reconstruct the averaged mechanical state (the full stress tensor) of each cell according to

$$\begin{array}{c} \sigma =\frac{1}{V}\int_{\Omega_{V}}\left(r-r_{0}\right)⊗T\left(r\right)\;dS \end{array}$$

where *V* and **r**_0_ are the volume and the centroid of the cell region Ω_*V*_, respectively, and **T**(**r**) is the traction at location **r**. See Fig 8. Physically speaking, the cell’s stress tensor, ***σ***, is obtained by integrating the traction **T**(**r**), force per area, acting on every point, **r**, on the cell membranes averaged by volume. The three orthogonal principal axes of the stress tensor point in the directions that the cell is shear-free, and the eigenvalues quantify the uniaxial tension along each principle direction. In Fig 8, we plot the principle axes of stress tensors on each cell, where the lengths of the axes show the relative magnitude of the tensile stress in the corresponding direction. One can see that the ABpl and ABpr cells are exposed to high levels of stress and stress anisotropy. See S5 and S6 Movies for more details of the stresses in both 7-cell-embryo and 12-cell-embryo. Similarly we can also reconstruct the shape tensor of each cell to quantify its relative size compared to other cells and its shape anisotropy. The shape tensor is computed by rescaling the moment of inertia tensor by volume:

$$\begin{array}{c} \tau =\frac{1}{V}\int_{\Omega_{V}}\left(r-r_{0}\right)⊗\left(r-r_{0}\right)\;dS. \end{array}$$

The three orthogonal principal axes of the shape tensor resemble the principal axes of an ellipsoid and the ratios between the eigenvalues quantify the shape anisotropy of the cell. In summary, here we have inferred forces in both 7-cell-embryo and 12-cell-embryo and have visualized their mechanical state accordingly.

### Quantitative assessment of errors

Here we present a sensitivity and reproducibility analysis of the proposed schemes. Errors in the inferred forces from the equilibrium solution arise from four main sources. First, noise can be introduced due to shot noise during image acquisition. Second, noise can be introduced via our segmentation protocol. Third, errors might be accrued by our modeling assumption were they to inaccurately represent the mechanical state of the embryo. Fourth, our method solves an overdetermined system, and as such not every balance relation can be fully satisfied. Below, we assess the errors of our scheme due to the overdetermined nature of our system of equations. In *Sensitivity analysis and reproducibility of protocol*, we discuss the robustness of our method to noise from the first two steps. The potential errors due to our simplifying mechanical assumptions will be tested via a correlation analysis of the myosin membrane density in a future study.

**Fig 9:**
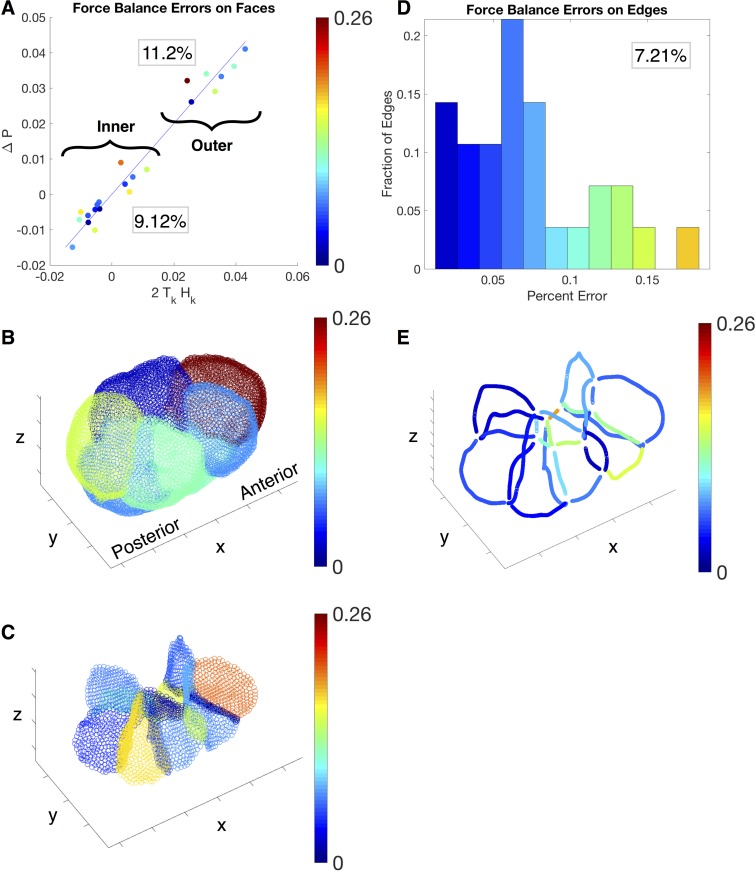
Error plots for inferred forces. Plot of the errors in the force balance relations on the membranes (A-C) and junctions (D-E). In A, we show errors in Eq (1) by plotting 2*T*_*k*_*H*_*k*_ against Δ*P* for each face in the scatter plot. The two clusters correspond to the inner and outer membranes, with their respective average percent errors in the box. Errors for the faces are portrayed as a heat map on the embryo, with outer membranes in B and inner membranes in C. In D, we plot the percent error equations, (2) and (3), as a histogram, with the average percent errors in the box. The percent errors of edges are portrayed as a heat map on the edge junctions (E), where the heat map corresponds to the heat map in the histogram (D).

We quantify the relative errors on the membranes and junctions separately. On each membrane, the absolute error is defined as the residual of the Young-Laplace relation (1). The relative error is obtained by (*P*_*i*_ − *P*_*j*_ − 2 ⋅ *H*_*k*_ ⋅ *T*_*k*_)/(|*P*_*i*_| + |*P*_*j*_| + |2 ⋅ *H*_*k*_ ⋅ *T*_*k*_|)—the residual of Eq (1) divided by the magnitude sum of each term. A scatter-plot of the left and right hand side of Eq (1) is reproduced in Fig 9(A). The two clusters in the scatter plot correspond to the force balance equations on the outer (Fig 9(B)) and inner membranes (Fig 9(C)). The clustering can be explained by the fact that the outer membranes have higher mean curvatures on average than the inner membranes. The colors of the points visualize the magnitude of the relative errors, which is also plotted in the same color code on the outer and inner membranes below. We note that the largest errors are concentrated on the anterior outer and inner membranes. The average relative error for the outer membranes is 11.2% and for the inner membranes is 9.12%.

On each junction, the force balance is described by Eqs (2) and (3) in the two orthogonal directions. We define the absolute force balance error by the magnitude of the residual vector from the two equations. The relative error is then obtained by rescaling the absolute error by the average total forces among all the junctions, where the total force on each junction is the summation of the three inferred tensions. We plot the errors among all the junctions both in the histogram (Fig 9(D)) and in the heat map (Fig 9(E)). We note that the largest error occurs on the shortest outer junction. The average relative error over the junctions is 7.21%.

### Sensitivity analysis and reproducibility of protocol

**Fig 10:**
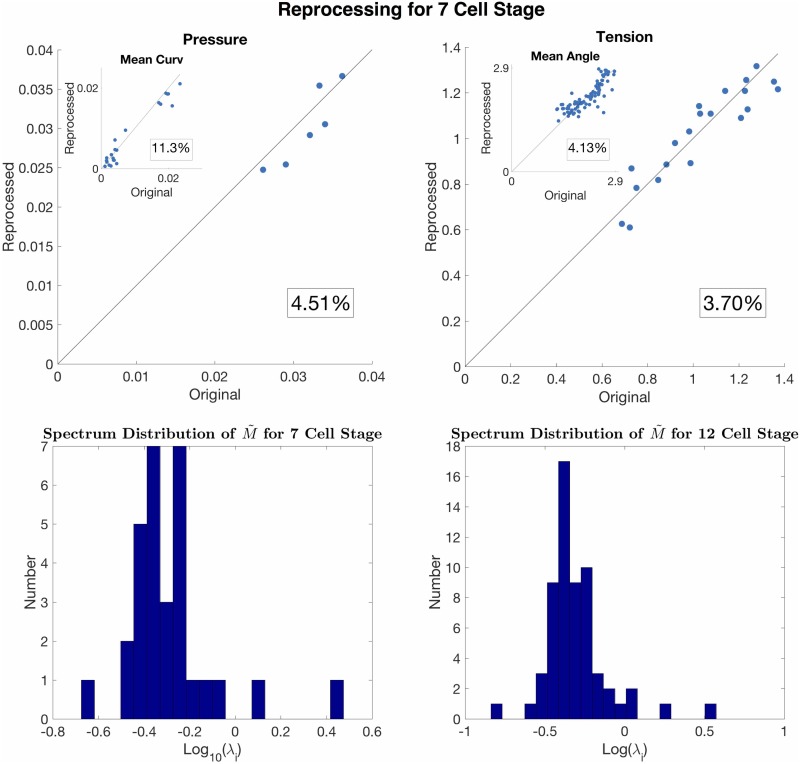
Sensitivity analysis and reproducibility. Plot of the error in the reprocessing at the 7 cell stage (top row). Scatter plots of original value against the reprocessed values, for the inferred pressure, tension, mean curvature, and mean angles. The percent error for each value is boxed. The eigenvalues of $\tilde{M}$ are plotted on a log-scale histogram (bottom row) with the 7-cell stage on the left and 12-cell stage on the right.

Here we perform sensitivity analysis to estimate the reliability of the results subject to noise. Under small perturbation of the coefficient matrix **M** + *δ***M** and **b** + *δ***b**, we can look at the spectrum (i.e., the set of eigenvalues *λ*_*i*_’s, *i* = 1, 2, …, *n*_*m*_ + *n*_*c*_ + 1) of the pseudoinverse $\tilde{M}={\left(M^{T}M\right)}^{-1}M^{T}$ to estimate the sensitivity of **x** + *δ***x** to *δ***M** and *δ***b**. Large *λ*_*i*_ > 1 indicates sensitivity to perturbations. We perform a sensitivity analysis on the $\tilde{M}$ of a 7-cell embryo and a 12-cell embryo, demonstrating that most of the eigenvalues are smaller than 1. (See the spectrum distribution in Fig 10) Interestingly, the eigenvector corresponding to the largest eigenvalue is in the direction of constant pressures and zero tensions. Since we are looking at the pressure difference from the exterior pressure, this perturbation mode does not contaminate the result. To check the reproducibility of our workflow, we reconstruct a grayscale image of the membranes from the smoothed point cloud by 1) generating a binarized image by rounding-off the positions of the point cloud to the nearest voxels, and 2) diffusing the membrane voxels 3-voxel-distances away followed by a linear decrease of the intensity value. This effectively generates a perturbed image with the same intensity profile. We run our workflow on the augmented data and find the relative error for the 7-cell-embryo to be below 5%. See the inferred pressure and tension using reprocessed data in Fig 10 vs forces using the original data.

## Discussion

In this study we have presented a morphological reconstruction scheme that forms the basis of a force inference method for analyzing the geometric and mechanical features of worm embryonic development. The first main contribution of our work is our image segmentation procedure, which provides high-resolution 3-dimensional reconstruction of the embryo. The accurate recovery of cellular geometry not only facilitates our 3-dimensional force inference scheme, but also has many other practical applications. For example, an accurate membrane recognition permits quantitative measurements of density of fluorescently tagged membrane-bound proteins. The second main contribution of our work is the development of the 3-dimensional force balance scheme, which incorporates interactions between cellular pressures and tensions of all cells simultaneously. This work differs from prior work by Fujita et al. [23] on worm embryos, which infer forces of the *C. elegans* 2-cell embryo. In particular, Fujita et al. infer tensions and pressures in a step-wise manner, while the scheme presented in this study solves for the cellular pressures and membrane tensions together. Including the traction from the eggshell is not included in our analysis, and is the subject of future work.

Our model makes several simplifying assumptions, which we now evaluate in turn. 1) Our inference scheme based on the Young-Laplace equations on membranes employs the assumption that tension is uniform and isotropic on each membrane. This assumption is not suitable when the curvature varies significantly across a single membrane. An extension of the method to account for anisotropic membrane tensions would require detection of the 2 principle curvatures of a membrane, and is the subject of a future study [24]. Note that a model that assumes isotropy infers the average of the direction dependent tensions on a membrane. Additionally, we have ignored any contribution due to bending—an elastic response. To this end, laser ablation experiments on membranes at the one cell stage [25] demonstrate the instantaneous retraction of membrances, instead of a flattening. Physically speaking, this suggests that the membrane tends to minimize surface area as opposed to curvatures—hinting that the contribution of elastic/bending effects is small. Statistical analysis of the spectra of membrane fluctuations would falsify our assumptions. 2) We assume that the traction forces between the eggshell and outer membranes are negligible [23]. To account for this possible source of external traction requires information regarding membrane contact points with the eggshell and information regarding the exerted traction forces. Neither of this is supplied by imaging data but is an interesting direction to pursue. 3) Aiming to make as few material/constitutive assumptions as possible, we have ignored dissipative forces in our model owing to the generic separation of timescales between relaxation following laser ablation and in vivo dynamics. Were dissipative aspects to be included, detailed information pertaining to velocities and course-grained, likely time-dependent material properties, would be required [8, 9, 12]. In its absence, our model should be thought of as an approximation, giving us access to the dominant contributions to membrane tensions and cellular pressures. The utility of simplifying assumptions is to give access to mechanical forces with as few directly measured parameters, such as time and space dependent viscosity.

This study fails to address several important aspects, which we now outline and are the subject of future studies. 1) Analysis of predicted tensions and pressure across biological replicates, wildtype worms at similar stages of development, is required before we have sufficient confidence in the measurement. 2) A correlation-based analysis of predicted membrane tensions and the measured myosin-density on membranes is required before we have confidence in our assumptions and methodology. 3) A study of whether predicted tensions predict the order of magnitude of laser ablation data is required. 4) Cells do move, and hence at some point confronting the velocity data in a principled manner is required. Does accounting for velocity as outlined in the VFM approach produce better correlations between predicted tensions and measured myosin density data? 5) The ability to pick up alterations in the mechanical state following cell specific, or embryo wide, perturbations of the activity state of myosin is required before we have confidence in the proposed methods. 6) If the method passes the above tests then looking at the temporal dynamics of the mechanical stress tensors will be revealing, particular during cell-division and rearrangement events. Do changes in the mechanical state of cells correlate with the onset/termination of key signaling/inductive events in the embryo? Does the method fail to predict myosin levels and ablation data at particular stages of development, and why? We anticipate that as cells get smaller in the embryo, fewer pixels span a membrane, which naturally will effect the quality of the inference.

More broadly speaking, we anticipate that force inference schemes will compliment the molecular tools under development to give us insight into morphogenesis. While FRET-based reporters, for example, give access to molecular level forces, connecting them to the processes of cell shape change and cell movement will require a model for how the two very different scales are connected. Force inference schemes on the other hand give insight into the forces that control gross cell shape and cell movement features but lack molecular insights. We anticipate that it will be combination of the aforementioned tools that will drive progress in the field.

## Supporting information

S1 Fig Reconstruction and force inference on two-cell systems. In different configurations where the ratio between the two cell pressures changes, curvatures of the three membranes and the three angles between the three membrane faces are reconstructed in different resolutions (*L* = 10, 20, 40). (**A**) shows relative and absolute errors between the reconstructed and the true curvature of the membrane face 1, one of the major membranes. (**B**) shows relative and absolute errors between the reconstructed and the true curvature of the membrane face 3, the interfacial membrane. (**C**) shows the sum of errors between the reconstructed and true angles divided by 2*π*, where the inset shows the error of the exterior angle between the two major membranes. (**D**) shows sum of errors between the inferred and true tension, where the inset plots the inferred pressure ratios vs the true pressure ratios. (**E**) shows residual of the force balance due to Laplace relation and (**F**) shows the residual of the force balance in the direction coplanar with circular junction.
(PDF)

S1 Text Supporting information. (PDF)

S1 Table Parameter Table. (PDF)

S1 Movie Movie 1. The heat map of average membrane mean curvatures for a 7-cell-stage embryo.
(AVI)

S2 Movie Movie 2. The heat map of average membrane mean curvatures for a 12-cell-stage embryo.
(AVI)

S3 Movie Movie 3. The heat map of average inferred tension and pressure for a 7-cell-stage embryo.
(AVI)

S4 Movie Movie 4. The heat map of average inferred tension and pressure for a 12-cell-stage embryo.
(AVI)

S5 Movie Movie 5. The cell-based stress distribution in a 7-cell-stage embryo.
(AVI)

S6 Movie Movie 6. The cell-based stress distribution in a 12-cell-stage embryo.
(AVI)
