# Supplementary material for: A scheme for 3-dimensional morphological reconstruction and force inference in the early C. elegans embryo
Source: PLoS One. 2018 Jul 10;13(7):e0199151. doi: 10.1371/journal.pone.0199151 (PMC6038995; doi:10.1371/journal.pone.0199151)
Supplement: S1 Fig — In different configurations where the ratio between the two cell pressures changes, curvatures of the three membranes and the three angles between the three membrane faces are reconstructed in different resolutions (L = 10, 20, 40). (A) shows relative and absolute errors between the reconstructed and the true curvature of the membrane face 1, one of the major membranes. (B) shows relative and absolute errors between the reconstructed and the true curvature of the membrane face 3, the interfacial membrane. (C) shows the sum of errors between the reconstructed and true angles divided by 2π, where the inset shows the error of the exterior angle between the two major membranes. (D) shows sum of errors between the inferred and true tension, where the inset plots the inferred pressure ratios vs the true pressure ratios. (E) shows residual of the force balance due to Laplace relation and (F) shows the residual of the force balance in the direction coplanar with circular junction. (PDF) [file pone.0199151.s001.pdf]

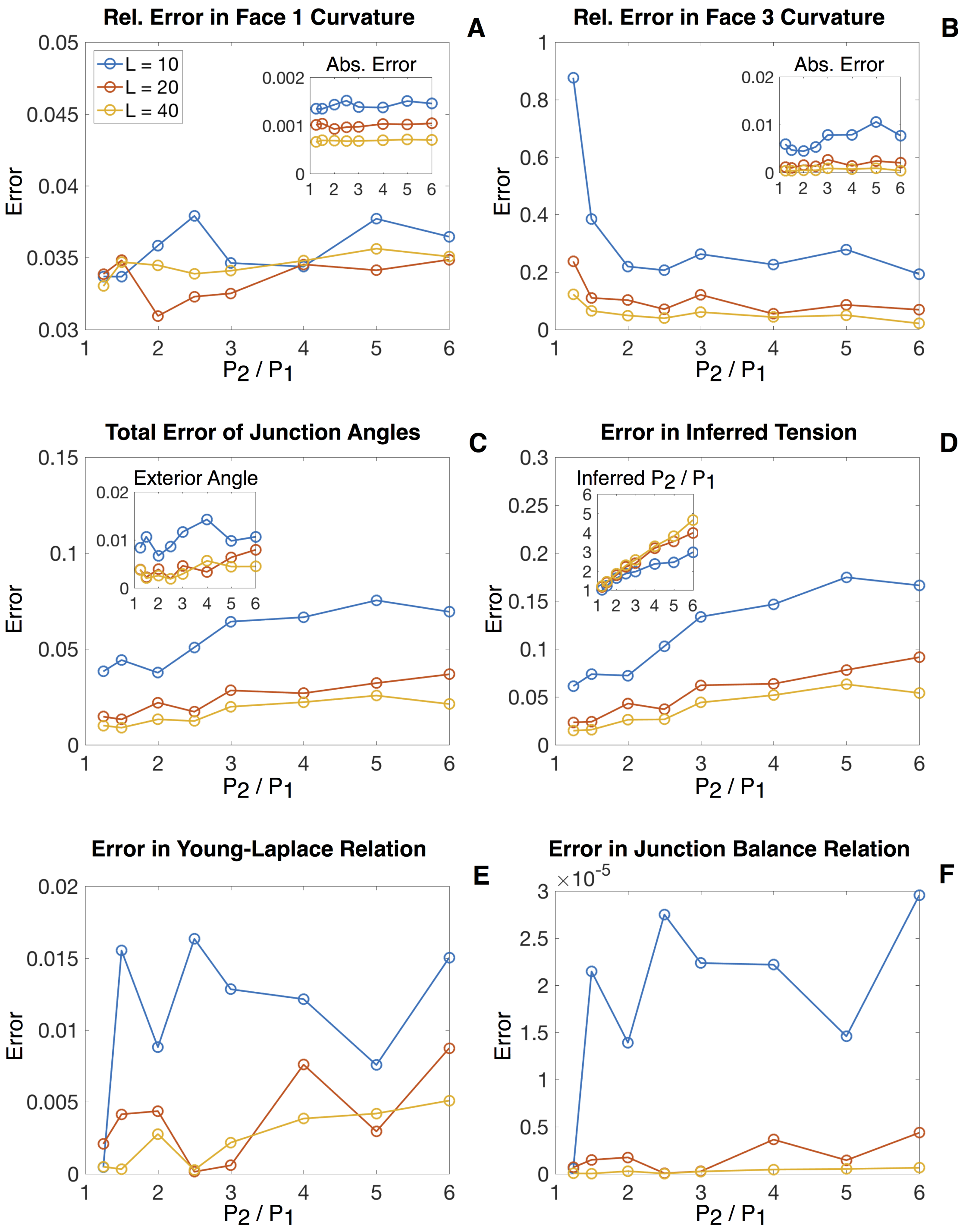

Figure 1: Reconstruction and force inference on two-cell systems
In different configurations where the ratio between the two cell pressures changes, curvatures of the three membranes and the three angles between the three membrane faces are reconstructed in different resolutions ($L = 10, 20, 40$). (**A**) shows relative and absolute errors between the reconstructed and the true curvature of the membrane face 1, one of the major membranes. (**B**) shows relative and absolute errors between the reconstructed and the true curvature of the membrane face 3, the interfacial membrane. (**C**) shows the sum of errors between the reconstructed and true angles divided by 2π, where the inset shows the error of the exterior angle between the two major membranes. (**D**) shows sum of errors between the inferred and true tension, where the inset plots the inferred pressure ratios vs the true pressure ratios. (**E**) shows residual of the force balance due to Laplace relation and (**F**) shows the residual of the force balance in the direction coplanar with circular junction.
