# Supplementary material for: A scheme for 3-dimensional morphological reconstruction and force inference in the early C. elegans embryo
Source: PLoS One. 2018 Jul 10;13(7):e0199151. doi: 10.1371/journal.pone.0199151 (PMC6038995; doi:10.1371/journal.pone.0199151)
Supplement: S1 Text — (PDF) [file pone.0199151.s002.pdf]

Supporting information

June 27, 2018

Details in the reconstruction of the morphology

We start the morphological reconstruction by using standard machine learning image analysis software (*Ilastik*) to generate a probability map, evaluating the likelihood of a voxel point to be on the membrane or in the cytoplasm (or the perivitelline space exterior to the embryo), trained by grayscale images of the plasma membranes at different time points. We take the data into *MATLAB* and threshold the membrane probability map by p_h (see S1 Table for the values), considering values below p_h as 0. We then dilate the membrane probability map on each voxel with a ball of radius r_h, removing small regions on the membrane that is preconsidered as cytoplasmic regions by (*Ilastik*) followed by an erosion of the dilated map with balls of radii r_h, preserving the original thickness of the membrane – i.e., the equivalent to morphological closing. In addition, we have identified and removed connected cytoplasm regions with size fewer than V_{min} voxels red to prevent spurious seeding of cells in the watershed segmentation. We then perform a watershed transformation on the probability map to obtain a classification of the voxels into cells, separated by a one voxel thick representation of the membrane. The membrane can be segmented by dilating adjacent cells one voxel and retrieving their intersection. The edges can then be retrieved by dilating adjacent membrane faces one voxel and selecting the intersection. At last, the pipeline described above give rises to a data structure with cells, membrane faces and edge junctions and their connectivities.

Mathematical concepts relevant to the geometry of membranes

A membrane is topologically a surface embedded in the three-dimensional Euclidean space ($\mathbf{R}^3$). The mean curvature at a point p on the surface S is an invariant describing how the surface is bent in the $\mathbf{R}^3$. Here we summarize only relevant concepts from differential geometry, to clarify our procedure in computing the mean curvature in our work. Mean curvature is the mean between two principal normal curvatures. In the following, we explain the concept of curvature, the normal curvature, the principal curvatures and finally the mean curvature. We describe how to compute the mean curvature in the end.

Curvature of a curve vs normal curvature of a surface

Given a curve $\mathbf{r}(s)$ embedded in $\mathbf{R}^3$ where s is the arc length along the curve, at a point p along the curve, the unit tangent vector is given as $\mathbf{r}'(s)|_{s=p}$. Then $\mathbf{r}''(s)$ is the rate of the change of unit tangent vector along s. We define the principal normal by

$$\mathbf{n}_s(s) = \frac{\mathbf{r}''(s)}{||\mathbf{r}''(s)||}. \tag{1}$$

The curvature of $\mathbf{r}(s)$ is defined as

$$\kappa(s) = ||\mathbf{r}''(s)||. \tag{2}$$

It is the rate of the change of tangent along the principal normal - $\mathbf{r}''(s) = \kappa(s)\mathbf{n}_s(s)$. Now, given a curve $\mathbf{r}(s) = \mathbf{r}(u(s), v(s))$ on a surface $\mathbf{r}(u, v)$, we can decompose $\mathbf{r}''(s)$ by

$$\mathbf{r}''(s) = \kappa_n(s)\mathbf{n}(s) + \kappa_g(s)\mathbf{n}(s) \times \mathbf{r}'(s), \tag{3}$$

where $\mathbf{n}(s)$ is the surface normal, orthogonal to tangent vectors on the surface in all directions. $\kappa_n(s)$ is called the normal curvature and $\kappa_g(s)$ is called the geodesic curvature. What is interesting is that $\kappa_n(s) = \mathbf{r}''(s) \cdot \mathbf{n}(s) = -\mathbf{r}'(s) \cdot \mathbf{n}'(s)$, only depends $\mathbf{r}'(s)$ and $\mathbf{n}'(s)$, respectively the unit tangent vector and the rate of the change of the surface normal. It measures how is the surface bent, a property of the surface, instead of a curve on the surface. Notice that $\mathbf{n}'(s)$ and $\mathbf{r}'(s)$ are both in the tangent plane of the surface S at p, defined as T_pS. The explanation is in the following subsection.

The Weingarten map, the principal curvatures and the mean curvature

The Weingarten map W_p is a unique linear map in T_pS and can be determined by

$$-(\mathbf{n}_u, \mathbf{n}_v)^T = W_p(\mathbf{r}_u, \mathbf{r}_v)^T. \tag{4}$$

So we also realize $-\mathbf{n}'(s) = W_p\mathbf{r}'(s)$ and $\kappa_n = \mathbf{r}'(s) \cdot W_p\mathbf{r}'(s)$. Notice $-\mathbf{n}_u$ and $-\mathbf{n}_v$ are in T_pS since $-\mathbf{n}_u \cdot \mathbf{n} = 0$ and $-\mathbf{n}_v \cdot \mathbf{n} = 0$. Given $\{\mathbf{r}_u, \mathbf{r}_v\}$ as the basis, W_p is a 2×2 matrix. Since it is symmetric (shown later), there is always a pair of real eigenvalues κ_1 and κ_2 with the corresponding basis $\hat{\mathbf{t}}_1$ and $\hat{\mathbf{t}}_2$ that satisfies

$$W_p(\hat{\mathbf{t}}_i) = \kappa_i\hat{\mathbf{t}}_i, i = 1, 2 \tag{5}$$

Notice κ_i is the normal curvature in the direction of $\hat{\mathbf{t}}_i$. The pair of the normal curvatures are called the principal curvatures of the surface at p. The mean $\kappa_m = \frac{\kappa_1+\kappa_2}{2}$ is mean curvature, and the product $\kappa_g = \kappa_1\kappa_2$ is the Gaussian curvature. They are both invariants of W_p. κ_1 and κ_2 are the eigenvalues of $W_p = [F_{II}F_I^{-1}]$ where

$$F_I = \begin{pmatrix} E & F \\ F & G \end{pmatrix} = \begin{pmatrix} \mathbf{r}_u \cdot \mathbf{r}_u & \mathbf{r}_u \cdot \mathbf{r}_v \\ \mathbf{r}_u \cdot \mathbf{r}_v & \mathbf{r}_v \cdot \mathbf{r}_v \end{pmatrix}, F_{II} = \begin{pmatrix} L & M \\ M & N \end{pmatrix} = \begin{pmatrix} -\mathbf{n}_u \cdot \mathbf{r}_u & -\mathbf{n}_u \cdot \mathbf{r}_v \\ -\mathbf{n}_v \cdot \mathbf{r}_u & -\mathbf{n}_v \cdot \mathbf{r}_v \end{pmatrix}. \tag{6}$$

One can see that W_p is symmetric.
