# Supplementary material for: A scheme for 3-dimensional morphological reconstruction and force inference in the early C. elegans embryo
Source: PLoS One. 2018 Jul 10;13(7):e0199151. doi: 10.1371/journal.pone.0199151 (PMC6038995; doi:10.1371/journal.pone.0199151)
Supplement: S1 Table — (PDF) [file pone.0199151.s003.pdf]

Parameter Table

Parameter	Value	Description
p_h	0.5	The membrane probability map threshold used in the reconstruction of the embryo.
r_h	3 (5)	The radius of the ball used in the morphological processes for the reconstruction of the embryo.
V_{min}	2000 (500)	The threshold for the minimum number of connected components used in the reconstruction of the embryo.
K_S	50	The number of nearest neighboring points used for membrane smoothing.
K_C	50 (800, 3200)	The number of nearest neighboring points used for curvature computation on the membrane.
d_T	8	The distance threshold in voxels from the other membranes from which a temporary junction point cloud is sampled.
K_J	240	The number of nearest neighboring points used to thin the temporary junction point cloud.
